# Which factors influence the ED length-of-stay after anterior shoulder dislocations: a retrospective chart review in 716 cases

**DOI:** 10.1186/s12873-020-00336-9

**Published:** 2020-05-20

**Authors:** Daan Schuur, David Baden, Martijn Roetman, Tom Boeije, Michael Burg, Nieke Mullaart-Jansen

**Affiliations:** 1 Dijklander Ziekenhuis, Maelsonstraat 3, 1624 NP Hoorn, the Netherlands; 2 Gloriantstraat 12-III, 1055 CT Amsterdam, Netherlands; 3 Diakonessenhuis Utrecht, Bosboomstraat 1, 3582 KE Utrecht, the Netherlands; 4 Flevoziekenhuis Almere, Hospitaalweg 1, 1315 RA Almere, the Netherlands; 5 University of California, San Francisco Fresno, 155N Fesno street, Fresno, CA 93701-2302 USA

**Keywords:** Shoulder dislocations, Glenohumeral joint, Reduction methods, Length of stay, Throughput times

## Abstract

**Background:**

Anterior shoulder dislocations (ASD) are commonly seen in Emergency Departments (ED). ED overcrowding is increasingly burdening many healthcare systems. Little is known about factors influencing ED length-of-stay (LOS) for ASD. This study defines the factors influencing ED LOS for ASD patients.

**Methods:**

Retrospective chart reviews were performed on all patients ≥12 years admitted with an anterior shoulder dislocation at two regional hospitals in the Netherlands between 2010 and 2016. The electronic patient records were reviewed for baseline patient characteristics, trauma mechanism, reduction methods, medication used, complications and the LOS at the ED. The main objective was determining factors influencing the LOS in patients with an anterior shoulder dislocation at the ED.

**Results:**

During the study period, 716 ASD occurred in 574 patients, 374 (65.2%) in males. There were 389 (54.3%) primary ASD; the remainder (327, 45.7%) were recurrent. Median LOS was 92 min (IQR 66 min), with a significantly shorter LOS in those with recurrent dislocations (*p* < 0.001), younger age group (*p* < 0.03) and in patients who received no medications in the ED (*p* < 0.001). Traction-countertraction and leverage techniques were associated with a significant more use of ED medication compared to other techniques. Although the use of more medication might suggest the LOS would be longer for these techniques, we did not find a significant difference between different reduction techniques and LOS.

**Conclusion:**

To our knowledge this study is the largest of its kind, demonstrating ED LOS in ASD patients is influenced by age, the need for medication and dislocation history, primary versus recurrent. Notably, we found that biomechanical reduction techniques, which are not primarily traction-countertraction or leverage techniques, e.g. scapular manipulation and Cunningham, were associated with less ED medication use. Further research is needed to define how reduction methods influence ED medication use, patient satisfaction and ED throughput times.

## Background

The highly mobile glenohumeral joint is the most commonly dislocated large joint with an annual incidence of 8–27 per 100,000 population [1–3]. The incidence of shoulder dislocations in the Netherlands is estimated to 5100 every year [3]. A vast majority of these dislocations are anterior, with the dislocated humeral head abnormally positioned anterior to the scapula. No consensus exists on the “best” anterior shoulder dislocation (ASD) reduction technique, nor does consensus exist for optimal medication use to effect ASD reduction.

Over 2000 years ago Hippocrates of Kos described a reduction method that is still used today. Many more reduction techniques have been described and are currently practiced. Techniques can be divided in three categories. Primarily traction-based, e.g. Hippocrates, Milch, primarily leverage-based, e.g. Kocher and so-called “biomechanical” techniques, e.g. scapular manipulation, FARES, modified Milch and Cunningham [4–8].

Worldwide, Emergency Departments are increasingly busy and overcrowded, leading to increased “door-to-provider” times and prolonged ED throughput times. The overall mean LOS for discharged patients in the Netherlands is 119 min [9–11]. A recent single centre Dutch hospital study showed that ED overcrowding occurred 30.8% of the time [10]. This is associated with negative patient outcomes, including treatment delays and a higher risk for adverse events [9–11].

ASD patients often require significant ED resources, such as medications, materials, monitoring and attention by ED personnel, further impacting and lengthening ED LOS and potentially effecting waiting times for other patients [6, 8, 12, 13].

The present literature contains little on factors determining ED LOS for ASD patients. We therefore initiated this study to determine which factors influence ED LOS for ASD patients. We postulated that reduction method, medication use, patient age and patient dislocation history (primary vs. recurrent) would influence LOS.

## Methods

Study data was collected from *Westfriesgasthuis (WFG)* in Hoorn*,* and the *Flevoziekenhuis (Flevo)* in Almere, the Netherlands. These two community EDs have approximately 50,000 visits a year combined. WFG is a level 2 trauma centre with 14 rooms at the ED and provides a training program for Emergency Medicine residents. Flevo is a level 3 trauma centre, with 16 rooms and without an Emergency Medicine residents training program. Ethics committee approval was obtained at both facilities.

Using the DBC-code, ‘Diagnose-Behandel-Combinatie’, meaning Diagnosis Treatment Combination, for “shoulder dislocation” we identified all the patients that visited the ED at WFG and Flevo Hospital between January 2010 and July 2016. The DBC is a unique code used in the Netherlands to identify certain combinations of diagnosis and treatment. In Flevo the orthopaedic and surgical department have a weekly schedule in supervising the ED and because of administrative issues we only included those patients that were seen for the orthopaedic department. The medical records were checked to only include the anterior shoulder dislocations, i.e. other directions of dislocation were excluded. Other exclusion criteria were age younger than 12 years, reduction before arrival at the ED and patients presenting after multitrauma or other health issues that made the ASD not the first priority of treatment. Repeated presentations of the same patient were recorded as different cases.

The patient characteristics were extracted from the electronic patient records (WFG; HiX version 6.0 HF55, manufactured by ChipSoft BV, Amsterdam and Flevo; i.s.h.med, manufactured by Cerner Corporation, North Kansas City). Data and statistical analysis was managed with IBM SPSS Statistics, version 20 for MacOSX. Continuous variables were compared for subgroups with the non-parametric Kruskall-Wallis H test and the Mann-Whitney U Test. The Pearson Chi-square test was used for evaluating differences between groups in categorical variables. Testing for normality was done with the Kolmogorov-Smirnov test. Numerical data are presented with median and interquartile range (IQR). As a cut-off point for significance, a *p*-value of 0.05 was used.

All medical record inconsistencies (e.g. radiograph interpretation discordance) were discussed by a panel consisting of the lead researcher, an emergency medicine resident and an emergency physician. Only panel consensus findings were used in these cases. The first author performed all data collection and 10% of this data was checked for reliability by the panel.

## Results

Records for 716 ASD occurring in 574 patients were available for analysis. Primary (first time) ASD numbered 389 (54.3%) and 327 (45.7%) were recurrent (more than one same-sided) ASD. Males sustained 475 (66.3%) of the ASD. There was a trimodal age distribution, with peaks at 21, 43 and 65 years.

**Fig. 1:**
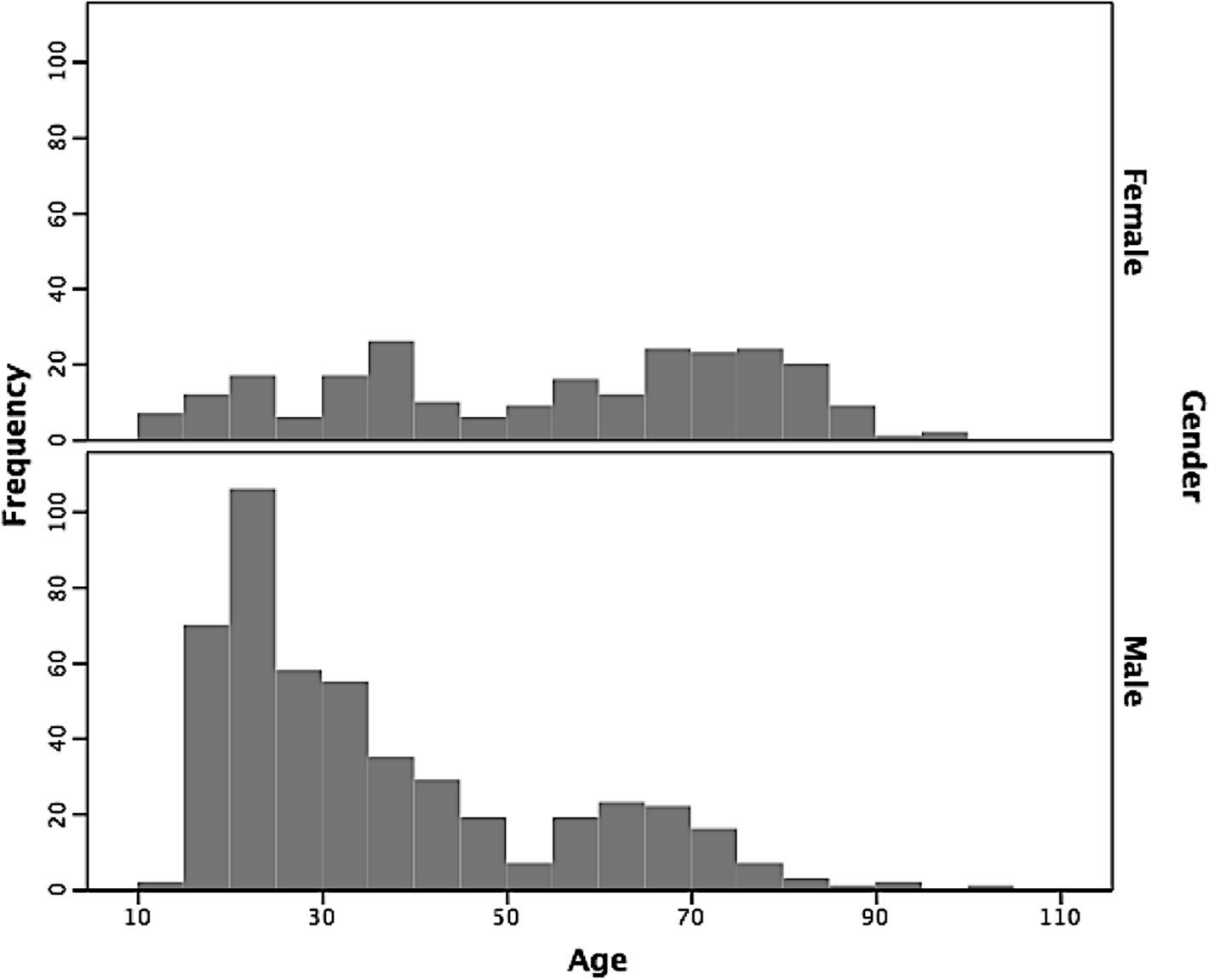
age distribution. Age distribution shows two peaks, with a median age for males of 30.0 yrs. and 59 yrs. for females

The overall median age was 35 years (IQR 39). The median age for males was 30 years (IQR 23) and 59 years (IQR 39) for females (Fig. 1). This difference in age was significant (*p* < 0.001). Recurrent ASD occurred in younger individuals (median age 28 years, IQR 17) compared with primary ASD (median age 48 years, IQR 38) (*p* < 0.001).

The most common ASD mechanisms were falls (34.1%), sports (25.0%) and non-traumatic causes (23.0%). Primary dislocations most commonly resulted from falls. Most recurrent dislocations were non-traumatic (Table 1).

**Table 1:** Mechanisms of dislocation regarding to being a primary or recurrent dislocation and regarding to age

Mechanism of dislocation	Median age (IQR)	Primary *N* = 389% of primary	Recurrent *N* = 327% of recurrent	Total *N* = 716% of total	< 35 yrs	36–55 yrs	56–75 yrs	> 75 yrs
**Non-traumatic**	28 (22)	14 3.6%	**151** **46.2%**	**165** **23.0%**	109 66.1%	25 15.2%	23 13.9%	8 4.8%
**Sports**	26 (17)	94 24.2%	85 26.0%	**179** **25.0%**	**130** **72.6%**	32 17.9%	15 8.4%	2 1.1%
**Traffic**	46 (44)	45 11.6%	9 2.8%	54 7.5%	21 38.9%	12 22.2%	16 29.6%	5 9.3%
**Fall**	61 (35)	**194** **49.9%**	50 15.3%	**244** **34.1%**	52 21.3%	50 20.5%	**98** **40.2%**	**44** **18.0%**
**Epileptic insult**	33 (11)	5 1.3%	8 2.4%	13 1.8%	9 69.2%	**3** **23.1%**	1 7.7%	0 0.0%
**Other**	26 (17)	37 9.5%	24 7.3%	61 8.5%	42 68.9%	12 19.7%	7 11.5%	0 0.0%
**Total**	35 (39)	389 54.3%	327 45.7%	716 100%	363 50.7%	134 18.7%	160 22.3%	59 8.2%

In fall-related ASD, more than half the patients were older than 55. In sports-related ASD nearly ¾ (72.6%) of the patients were under the age of 35 (Table 1). ASD due to falls occurred in significantly older patients compared with other dislocation causes, with a median age of 61 years (IQR 35) (*p* < 0.001).

Sports-related dislocations occurred in younger patients compared to non-traumatic causes (*p* = 0.011), traffic (*p* < 0.001), and falls (*p* < 0.001) with the median age being 26 years (IQR 17).

First attempt reduction methods are listed in Table 2, with the most popular being the Cunningham method and traction-countertraction methods (which includes the Hippocrates method and the Stimson technique).

**Table 2:** Reduction methods used

Reduction method;	Frequency	Percent
**Unknown**	363	50.7
**Cunningham**	114	15.9
**Traction-countertraction**	113	15.8
**Milch**	81	11.3
**Kocher**	22	3.1
**Scapular Manipulation**	17	2.4
**Other**	6	0.8
**Total**	716	100

There were a total of 360 associated injuries, occurring in 287 patients.

A Hill Sachs lesion was the most common associated local injury (198/716, 27.7%), followed by Bankart’s lesion (58/716, 8.1%) and greater tubercle fractures (45/716, 6.3%) (Table 3)*.* There was a significant difference in age with greater tubercle fractures (*p* < 0.001), subcapital humerus fractures (*p* = 0.015), nerve damage (*p* = 0.008) and damage to the rotator cuff muscles (*p* < 0.001), all being more common in the elderly. Although 52.0% of the Hill Sachs lesions occurred in patients under the age of 35, we did not find a significant difference in age (*p* = 0.253).

**Table 3:** Associated injuries

	N	% of patients
**No associated injuries**	429	59.9
**Hills Sachs lesion**	198	27.7
**Bankart’s lesion**	58	8.1
**Tuberculum majus fracture**	45	6.3
**Subcapital humerus fracture**	4	0.6
**Nerve damage**	25	3.5
**Vascular damage**	0	0.0
**Muscle rupture**	30	4.2
**Total number of associated injuries**	360	
**Total patients with associated injuries**	287	40.1
**Total patients**	716	100

Reduction methods based on traction-countertraction and leverage were used in 135 of the cases. Biomechanical techniques were used in 212 cases. In 369 (51.5%) cases the reduction method used was not specified.

Medication use (opiates, benzodiazepines, procedural sedation and analgesia, intra-articular lidocaine) differed significantly with reduction methods used. In 33% (*n* = 70) of biomechanical reductions, no medications were used. When traction-countertraction or leverage techniques were employed, a non-medication approach occurred in only 16.3% (*n* = 22) of cases (*p* < 0.01).

The median length-of-stay (LOS) was 92 min (IQR 66). The LOS was not obtainable from 16 charts. LOS differed significantly between recurrent and primary ASD (*p* < 0.001), the median LOS being 78 min (IQR 54.5) for recurrent dislocations and 102 min (IQR 68) for primary dislocations.

LOS differed significantly between patients given ED medications to facilitate reduction and those not given medications, with a median LOS of 99 min (IQR 67) for the medicated group compared to a LOS of 61 min (IQR 60) for the un-medicated group (*p* < 0.001). There was also a significant difference in LOS for those who received procedural sedation and analgesia, median 139 min (IQR 89) and those who received other kinds of analgesia, median 90 min (IQR 54.0) (*p* < 0.001) (Table 4).

**Table 4:** Length of Stay in minutes

	N	Median (IQR)
**Total**	700	92.0 (65.75)
**Recurrent dislocation**	321	78.0 (54.5)
**Primary dislocation**	379	102.0 (68.0)
**Medication**		
**No prescription drugs administered**	159	61.0 (60.0)
**Any kind of prescription drugs administered**	541	99.0 (67.0)
**Medication administered (excl. PSA)**	396	90.0 (54.0)
**PSA administered**	143	139.0 (89.0)
**Age**		
**< 35 yrs**	249	88.0 (57.0)
**36–55 yrs**	104	92.5 (62.8)
**56–75 yrs**	137	110.0 (79.5)
**> 75 yrs**	51	134.0 (100.0)

Prescription drugs; i.e. opiates, benzodiazepines, intra-articular lidocaine, or PSA*,* PSA; Procedural Sedation and Analgesia

We classified the cases in age-groups to determine the effects of age (< 35, 36–55, 56–75 and > 75 yrs). The LOS was longer for the more elderly compared to the younger ones. This difference was significant in both the overall population as in the recurrent or primary dislocation groups (Table 4).

When we compared the LOS for the different kind of reduction methods used, we did not find any significant differences (*p* > 0.05). The ED LOS was not influenced by the ASD reduction method used.

## Discussion

We found a trimodal age distribution with the peaks at 21, 43 and 65 years. The median length of stay was 92 min (IQR 66) and was longer for the elderly, in those with primary dislocations and in those cases in which medication was administered. Other literature generally describes a bimodal age distribution [14–16]. In younger patients, sports were the main cause leading to dislocation, while in elderly it mostly occurred due to a fall.

Our incidence of Hill Sachs lesions, Bankart’s lesions and greater tubercle fractures were similar to other series [14, 17–19]. Our study recorded a 3.5% nerve injury incidence, which is much lower than the reported incidence of 12–21% in other studies.^5,29,33^ This could be due to underreporting or our focus on ED records only, since some nerve injury presentations are delayed. Some of our patients may have had follow up at other hospitals also leading to missing data on associated injuries.

ASD is a stressful painful experience. It has been suggested that successful reduction depends primarily on local muscle relaxation [12, 20]. For this reason, analgesic, anaesthetic or sedating agents are often administered. Procedural sedation and analgesia (PSA) effectively provides muscular relaxation and pain relief but confers a substantial side effect risk and generally requires more resources and prolonged ED lengths of stay [6, 12, 21, 22]. The administration of PSA is related to a longer LOS but also with a higher risk of complications compared to other kinds of medication [6, 12, 21]. In our series we found that the LOS for those who did not receive any kind of medication was 61 min compared to 99 min for the ones who got any kind of medication. Patients receiving PSA had a significantly longer ED LOS, over 1 h longer when compared to un-medicated patients and more than 40 min longer when compared to those who received other kinds of medication, not being PSA.

The availability to make X-ray’s at the ED is an important factor for the LOS. Both the WFG as the Flevo have a dedicated room for making X-ray’s for the emergency department, but the radiological technician making the x-rays can also be busy with operating the CT-scanner during evening and night shifts. So maybe hospitals with more capacity, facilities or staff might provide shorter LOS, but some delay will always be a part of the day to day routine of an emergency department.

## Limitations

Our study is a retrospective chart review, therefore uncontrolled. Patient evaluation and treatment was performed according to individual physician preference. Some data, like individual physician experience, physician workload, ED census at a given time and a variety of other information which may impact physician decision making or approach, are not obtainable from electronic health records and are therefore not taken into account. In the *WFG* there is an Emergency Medicine residency training program, while the *Flevo* does not have this program. This could have led to a different treatment approach or more delay in treatment. For the *Flevo* we only included the patients when the orthopaedic department was on call (they alternate every other week with the surgical department), leading to an incomplete view of the patients that visited the ED.

Patient characteristics were abstracted from electronic health records, which are not designed for research purposes. Factors like patient build, comorbidities and time from presentation to reduction, all of which could influence LOS, could not be retrieved from available health records [21, 23]. Some data, like trauma mechanism and reduction methods used had to be abstracted from written medical history, which could have resulted in incomplete and inconsistent registration, which could skew our results. In 61/716 (8.5%) records the mechanism of trauma could not be fully defined.

We found no significant correlation between reduction methods used and LOS, although the reduction method was unknown in 51.5% of cases. It might be that only successful reduction methods were recorded and failed reduction methods omitted. The number of attempts and the time needed for the reduction itself were not recorded either.

## Conclusion

To our knowledge this is the largest chart review focused on the length of stay at the emergency department (ED) for patients with an anterior shoulder dislocation.

In our study the median length of stay at the ED of patients with an anterior shoulder dislocation was 92 (IQR 66) minutes. An increased length of stay was found in patients with a primary dislocation, elderly patients and in those given any kind of medication before or during reduction. The administration of medication could lengthen the stay with more than 30 min.

We also found that medications were much more likely to be given to those undergoing traction-countertraction or leverage-based techniques, such as Hippocrates and Kocher, compared to biomechanical reduction techniques, such as scapular manipulation, Cunningham and (modified) Milch (84 vs 67%).

ED crowding and resultant prolonged lengths-of-stay are increasing worldwide. In case of the commonly-seen anterior shoulder dislocation little can be done about patient characteristics. Our results suggest that ED LOS in this patient category can be significantly reduced if anterior shoulder dislocations are treated with reduction techniques that minimize medication use, especially procedural sedation and analgesia.

Development of an evidence based algorithmic approach to ASD reduction starting with, and focused on, pain-minimizing techniques is warranted.

## Data Availability

Please contact author for data requests.

## Abbreviations

ED: Emergency department
ASD: Anterior shoulder dislocation
LOS: Length of stay
IQR: Interquartile range
PSA: Procedural sedation and analgesia
WFG: Westfriesgasthuis, hospital in Hoorn, the Netherlands
Flevo: Flevoziekenhuis, hospital in Almere, the Netherlands

## References

[1] Krøner K, Lind T, Jensen J (1989). The epidemiology of shoulder dislocations. Arch Orthop Trauma Surg.

[2] Simonet WT, Melton LJ, Cofield RH (1984). Incidence of anterior shoulder dislocation in Olmsted County, Minnesota. Clin Orthop Relat Res.

[3] te Slaa RL, Bron C, den Hollander H, et al. Acute primaire schouderluxatie: diagnostiek en behandeling. Alphen aan den Rijn: van Zuiden Communications B.V, 2005.

[4] te Slaa RL, Wijffels MPJM, Marti RK (2003). Questionnaire reveals variations in the management of acute first time shoulder dislocations in the Netherlands. Eur J Emerg Med.

[5] Mattick A, Wyatt JP (2000). From Hippocrates to the Eskimo - a history of techniques used to reduce anterior dislocation of the shoulder. J R Coll Surg Edinb.

[6] Hendey GW (2015). Managing anterior shoulder dislocation. Ann Emerg Med Am Coll Emerg Phys.

[7] Cunningham N (2003). A new drug free technique for reducing anterior shoulder dislocations. Emerg Med.

[8] Riebel GD, McCabe JB (1991). Anterior shoulder dislocation: a review of reduction techniques. Am J Emerg Med.

[9] van der Linden C, Reijnen R, Derlet RW (2013). Emergency department crowding in the Netherlands: managers’ experiences. Int J Emerg Med.

[10] van der Linden N, van der Linden MC, Richards JR, et al. Effects of emergency department crowding on the delivery of timely care in an inner-city hospital in the Netherlands. Eur J Emerg Med. 2015;1. 10.1097/MEJ.0000000000000268 25831039

[11] Horwitz LI, Green J, Bradley EH (2010). United States emergency department performance on wait time and length of visit. Ann Emerg Med.

[12] Janitzky AA, Akyol C, Kesapli M (2015). Anterior shoulder dislocations in busy emergency departments: the external rotation without sedation and analgesia (ERWOSA) method may be the first choice for reduction. Medicine (Baltimore).

[13] Descamps MJL, Gwilym S, Weldon D (2007). Prospective audit of emergency department transit times associated with entonox analgesia for reduction of the acute, traumatic dislocated shoulder. Accid Emerg Nurs.

[14] te Slaa RL, Wijffels MPJM, Brand R (2004). The prognosis following acute primary glenohumeral dislocation. J Bone Joint Surg Br.

[15] Nordqvist A, Petersson CJ (1995). Incidence and causes of shoulder girdle injuries in an urban population. J Shoulder Elb Surg.

[16] Cutts S, Prempeh M, Drew S (2009). Anterior shoulder dislocation. Ann R Coll Surg Engl.

[17] Perron AD, Ingerski MS, Brady WJ (2003). Acute complications associated with shoulder dislocation at an academic emergency department. J Emerg Med.

[18] Pasila M, Jaroma H, Kiviluoto O (1978). Early complications of primary shoulder dislocations. Acta Orthop Scand.

[19] Hovelius L, Augustini BG, Fredin H (1996). Primary anterior dislocation of the shoulder in young patients. J bone Jt Surg.

[20] Jiang N, Hu YJ, Zhang KR, Zhang S, Yu B (2014). Intra-articular lidocaine versus intravenous analgesia and sedation for manual closed reduction of acute anterior shoulder dislocation: An updated meta-analysis. J Clin Anesth. Elsevier Inc.

[21] Kuhn JE (2006). Treating the initial anterior shoulder dislocation - an evidence-based medicine approach. Sports Med Arthrosc.

[22] Blaivas M, Adhikari S, Lander L (2011). A prospective comparison of procedural sedation and ultrasound-guided interscalene nerve block for shoulder reduction in the emergency department. Acad Emerg Med.

[23] Beattie TF, Steedman DJ, McGowan A, Robertson CE (1986). A comparison of the Milch and Kocher techniques for acute anterior dislocation of the shoulder. J Sports Med.

